# Rethinking stabilization policies; Including supply-side measures and entrepreneurial processes

**DOI:** 10.1007/s11187-021-00520-6

**Published:** 2021-08-03

**Authors:** Pontus Braunerhjelm

**Affiliations:** Swedish Entrepreneurship Forum, KTH Royal Institute of Technology and Blekinge Institute of Technology, Stockholm and Karlskrona, Sweden

**Keywords:** Crisis, Stabilization policies, Supply side, Incentives, Entrepreneurs, L26, O3, E32, E58, E62

## Abstract

Traditional macroeconomic stabilization policies seek to moderate swings in economic activity through measures that primarily augment aggregate demand. Such measures are, however, inadequate in mitigating the comprehensive effects of crisis such as the COVID-19, which affects both the demand and supply sides of the economy. Moreover, monetary policies are presently close to a liquidity trap combined with weakened transmission links to the real economy. Fiscal policies have been reactivated, albeit in an ad hoc and experimental manner. Based on a literature review and the policy responses following the COVID-19 crisis, the objective is to present a modified and extended framework for stabilization policies. In particular, the importance of microeconomic supply-side measures that promote entrepreneurial processes and knowledge-upgrading efforts are emphasized. Furthermore, a coherent realigning of policies at the micro- and macro-levels is argued to enhance the potential for long-term growth and to facilitate the restructuring of an economy that normally follows a crisis.

## Abstract (plain language summary)

The COVID-19 crisis makes traditional stabilization policies obsolete. Reinstate the market and redirect policy from interest rates and unconditional state support toward providing employees and firms with adequate knowledge for future challenges. Traditional crises policies seek to moderate swings in economic activity by primarily lowering interest rates and increase governmental expenditure to stimulate demand and economic activity. However, the effectiveness of both of these measures has been questioned, in particular, further reductions in already extremely low-interest rates. The present COVID-19 crisis has highlighted the importance of taking firms, entrepreneurs, trade, etc., into account, i.e., the supply side of the economy. It is argued that traditional policies should partly be replaced by measures targeting entrepreneurial processes, firm growth, innovation, and knowledge upgrading. Corporate taxes should be used to increase firms’ crisis resilience, increase investment, and encourage start-ups, while state support should be conditioned on employees engaging in knowledge upgrading. Hence, the main conclusion of this study is that such redirection of policies will more effectively level out swings in the business cycle, increase the potential for long-term growth, and make it easier for employees and firms to adjust to new economic conditions.

## Introduction^1^

In 2020, the world economy experienced one of its deepest economic downturns since at least the Great Depression. At that time, the downturn spiraled into an economic depression that lasted most of the 1930s. This time, it looks different, i.e., the severe recession in 2020 seems to be followed by a relatively swift recovery in 2021–2022. Uncertainty concerning the trajectory of the crisis is, however, genuine; the jury is still out. The global signs of economic recovery and optimism during summer and early autumn were hampered by an extensive and rapid second wave of the virus in the fourth quarter of 2020, followed by the third wave in February–March of 2021. The latter contained mutated and more contagious versions of the virus. Hence, cautiousness in predicting the future path of COVID-19 and its economic impacts is warranted.^2^

Irrespective of how the COVID-19 pandemic evolves, there are reasons to believe that it entails important lessons for how future crises may evolve and spread across countries and how to cope with them. Even though the integration of the global economy has slowed somewhat since the financial market crisis of 2008–2009, the world economy remains highly intertwined and susceptible to the transmission of shocks. Interlinked value-added production chains still account for approximately 70% of world trade, while the global population, migration flows, and the number of refugees all continue to increase.^3^ This suggests that future shocks could also originate in hitherto unknown causes—such as new pandemics or environmentally induced crises—and diffuse rapidly.

The COVID-19 crisis deviates from most former crises of the last century that can be traced to the financial and real estate sectors of the economy (Rheinhart & Rogoff, 2010). A typical crisis pattern is the building up of unsustainable debt among households and firms, speculation and animal spirits, bankruptcies, declining asset values, financial market turbulence, unemployment, and falling demand. The present crisis, however, took another route. First, it started with the disruption of the supply side of the economy. The fragmented and globally organized production chains that have characterized industrial production in recent decades collapsed in the initial phase of the crisis because of the enforced restrictions on mobility, travel, and trade. Second, these distortions were then transmitted to the demand side since, basically, a curfew was imposed in several countries, leading to initial decreases in employee income and the inability of firms to produce.^4^ Third, in comparison with previous crises, the COVID-19 crisis spread quickly—within just weeks or months—and it turned out to be considerably more pervasive in terms of countries, industries, firms, and individuals affected than the typical crisis. Such comprehensive crises are more likely to generate long-lasting negative effects on the economy if not mitigated through active policies and support measures (Gregory et al., 2020).

The crisis laid bare the weaknesses of the traditional macroeconomic toolbox to stabilize economic shocks, i.e., to smooth out swings in economic activity. This is evident from the unparalleled number of, and experimentally implemented, policy measures. Whereas emphasis previously has been on monetary policies, this crisis has seen not only a return to more traditional Keynesian (or neo-Keynesian) policies but also the adoption of a host of other and less conventional instruments. Previous concerns regarding budget deficits and sovereign debts, particularly among EU countries where most had already exceeded the stipulated constraints in the Maastricht agreement, vanished or were abolished.^5^ Rather, it was emphasized how low long-term interest rates constituted an opportunity to increase lending and engage in expansionary fiscal policies (Krugman, 2020).

Since expansionary monetary policies in the last decade have led to historically low, even negative, interest rates, their efficiency in stimulating demand has also withered.^6^ This provided further impetus for policymakers to shift toward fiscal policy measures such as initiating infrastructure projects (environmental and other), strengthening social security systems and automatic stabilizers, tax considerations, and a more general expansion of government expenditure. In addition, policies are, to a larger extent, characterized by microeconomic measures targeting industries, firms, and individuals directly rather than by stimulating aggregate demand through large-scale macroeconomic policies.^7^ Some of these measures have been used previously, while some are new, but their adequacy as stabilization policy tools needs to be further examined and evaluated.

Based on the experimental policy approach featuring the present crisis and the obvious need to reconsider or complement traditional policy measures, the objective of this article is to present a modified and more comprehensive stabilization policy framework to counter large-scale and pervasive crises. More specifically, one prime and two secondary issues will be pursued. First, the importance of including the supply side and coherently aligning macro-policies targeting aggregate demand with supply-side microeconomic measures will be emphasized. Second, I will argue that a properly designed and cohesive strategy to stabilize the economy is compatible with improving the prerequisites for long-term growth,^8^ in addition to facilitating structural adjustment of the economy.

An efficient transmission of policy measures targeting the aggregate level requires that the microeconomic structures of an economy are taken into account. Entrepreneurial processes, either within incumbents (intrapreneurship) or in the form of new ventures, will be critical in restructuring and restarting an economy. Incentives that foster knowledge upgrading related to digitization, sustainable production systems, and resilient internationalization structures constitute one crucial element to accomplish this transformation. Such measures will also serve to strengthen employees’ and the business sector’s competitiveness and deepen the knowledge base of the economy.

The most important supply-side policy instruments at the micro-level are claimed to be knowledge upgrading/accumulation, taxes, access to finance, labor market flexibility, competition, and insolvency institutions. The composition and level of implementation can be expected to vary across countries and regions. Some of the suggested proposals are short term and temporary (taxes, financing, training, and knowledge upgrading), aiming to bridge swings in the business cycle and prepare economic agents for a turnaround in economic activity. Others serve to enhance the efficiency of short-term policies in the somewhat longer run (functioning labor markets, competition, and insolvency institutions).

Moreover, since crises tend to be characterized by different phases, policies need to adapt over time. The immediate “whatever it takes” strategy in the COVID-19 crisis was appropriate for the first phase but is not necessarily adequate in the subsequent stages or if a similar shock appears in the future. An excessively extensive policy agenda of subsidies and support schemes risks stifling the necessary restructuring of the economy and hampering the introduction of new firms, new business models, and innovations, while instead nurturing so-called zombie companies.^9^ A flexible approach may be needed where strategies change according to the different stages of the crisis.

A number of important issues, for instance, related to sustainability aspects, fall outside the scope of this paper.^10^ Such aspects should be integrated into crisis policies; while my analysis does not in any way hinder that, how and through which means is not explicitly elaborated in this study. In the somewhat longer run, it may serve to create significant competitive advantages for businesses.^11^ Another area that is not considered refers to the tensions between rural and metropolitan regions, which may be affected by crises differently for several reasons. Rural areas often see a strong concentration of industrial production, whereas services are more spatially distributed (e.g., tourism), implying that the crisis strikes differently depending on the pattern of specialization.^12^

In what follows, I will first give a brief account of the policy responses to the crisis and the different support schemes introduced in the EU and a number of countries. The subsequent Sect. 3 discusses the strengths and weaknesses of traditional macroeconomic stabilization policies, how they evolved over time, and why they need to be redesigned and complemented with other measures. Section 4 presents how micro-level supply-side policies could be better realigned with macroeconomic measures to boost the effectiveness of stabilization policies. The final Sect. 5 concludes and summarizes.

## Background: policy measures in the first and second stages of the COVID-19 crisis

A wide array of policy measures have been undertaken in most countries to mitigate the COVID-19 crisis. The OECD (2020b) has carefully documented the support measures implemented at the national and regional levels. Similarly, the effects of the crisis have also been reported on an ongoing basis, mainly based on surveys conducted in a large number of countries. During the second quarter of 2020, basically all of these surveys revealed sharp falls in employment and turnover, financial difficulties, and an increase in closures and bankruptcies (Bartik et al., 2020). In an analysis of 17 countries, Gourinchas et al. (2020) conclude that the average failure rate is expected to increase by almost 10% during the crisis, with certain sectors being considerably harder hit (recreation, travel, etc.). However, during the following third quarter, which was characterized by a decline in the spread of the virus and a lifting of different lockdown measures, economic activities bounced back, albeit not enough to compensate for the earlier decline. Notably, including the fourth quarter, bankruptcies were lower in 2020 than in 2019, illustrating the extent of subsidies to firms.^13^

Regarding the extent of the decline in economic activity, the first estimates predicted a fall of approximately 7% in the EU (Eurostat, 2020a; European Commission, 2020b), which turned out to be quite accurate according to the preliminary statistics for 2020. The decline in global GDP was in the same range, around 5%. The most recent forecast by the International Labor Organization (ILO, 2021) points to global unemployment increasing by 1.1% paired with a decrease in labor force participation of 2.2%, while the WTO (2020) estimates that trade will diminish by 9–10% in 2020. Predictions of the economic outcome of the COVID-19 crisis have been volatile, mirroring the uncertainty surrounding the diffusion and the effects of the different mutations that have appeared.^14^ Presently, as the vaccine is rolled out, the optimistic view dominates.

The immediate policy response in the initial phase of the crisis was to implement monetary measures to ensure that markets were liquid, accompanied by the provision of state-guaranteed loans. These policies were extended by a number of less conventional measures at the industry and firm levels, which can be divided into the following categories: (i) general, i.e., covering all sectors and companies (e.g., tax deferrals); (ii) sector-specific measures (e.g., tourism); and (iii) those primarily targeted at SMEs, start-ups, and innovation (e.g., earmarked funding). In particular, SMEs have been the focus of several policy initiatives. The justification is that in most countries, these account for the majority of employment and value added and represent the largest number of enterprises. Moreover, they also have a weaker financial position and modest cash reserves, are less digitized, and generally have lower technical know-how than larger firms, while in a normal market situation assessed to have viable future prospects.

Measures to support companies—apart from limiting the spread of the pandemic—can be classified as policies to increase liquidity in the market, deferral of taxes and employer contributions, loan guarantees, financing (access to credit, cash benefits, a moratorium on repayments, and interest), furlough wages and employment subsidies, and, finally, structural measures directed toward competence strengthening, sustainability strategies, and innovation. The latter measures have, however, been relatively rare.

The World Bank claims that approximately 850 different policy instruments have been used (World Bank, 2020), the most common of which are summarized in Table 1, although they come in a plethora of varieties. The levels of these support schemes are historic at the same time as large differences prevail between countries, with possible long-run economic consequences (e.g., policies to increase R&D and foster innovations). It is also questionable whether these measures have had the intended effect and reached those most in need of support, issues that will be further elaborated in the policy section below.

**Table 1:** An overview of national policy measures categorized on specific policy areas, targets/recipients, and share of countries

**Targets**	National policies									
	Finance			Subsidies	Taxes	Labor	Innovation	R&D	Bankruptcy	Knowledge
	Loan guarantees	Direct cash transfers	Venture capital	Compensate for loss of revenue, premises, fixed costs, etc	Deferrals, temporary abolishment, extended deductions	Furloughs, subsidies	Funding	Funding, extended deduction	Moratorium	Training (digitization, teleworking, innovation, etc.)
General	Most			Most	Most	Most			Few	
Sectors/industries		Some		Some	Some	-	Some	Some		
SMEs		Some		Some	Some	Some	Some	Few	Few	Few
Start-ups			Few	Few	-		Few			
Unemployed		Few								Few

*Source:* OECD (2020b), World Bank (2020)

Note: The implemented policy measures primarily refer to OECD countries. The degree to which these are implemented varies across nations and a full account is not possible due to the large number of policy measures and differences in reporting them. “Most” means that a majority of countries are assessed to have undertaken policies within a specific policy area, whereas “some” refers to a large group of countries but far from all and “few” implies that a minority of countries have engaged in a certain policy. When a policy measure, e.g., subsidies, appears several times in a column, the interpretation is that policies have been implemented on a general level embracing all sectors of the economy but in addition there are also policies targeting specific industries, SMEs, etc.

Most of these policy measures remained in place during the second stage (third quarter 2020), even though some became more limited (e.g., the percentage share that furlough wages would cover), while others had more of a one-time character (e.g., cash injections to households and firms). Note that within the EU, these crisis efforts have been made possible by changing the state aid rules in March 2020, followed by further easing in April. These were recently prolonged until December 31, 2021.^15^

The EU has implemented extensive support measures, including the Corona Response Initiative in March 2020 (around 37 billion euros), followed by 28 billion euros from the EU’s structural funds. The most ambitious initiative is the EU’s Recovery and Resilience Fund of 750 billion euros, distributed between 390 billion euros in grants and 360 billion in loans. This corresponds to almost 5% of the EU’s GDP and is planned to be financed through a mix of emitting bonds, EU taxes (carbon dioxide, financial, and digital transactions), and green tariffs. The ECB (2020) has also taken action, starting with the Pandemic Emergency Purchase Program (PEPP) in March 2020, and announcing that further efforts may be needed to increase liquidity and keep interest rates down. Similarly, the European Investment Bank (EIB) increased its commitments as the crisis became deeper.

Additionally, at the country level, a number of policy initiatives have been implemented, varying in extent and coverage.^16^ For instance, in the USA, the Paycheck Protection Program (PPP) was introduced early to alleviate financial constraints for firms with fewer than 500 employees. Similarly, in March 2020, the CARES Act was passed, consisting of 2000 billion US dollars allocated between loans to large and small firms and direct cash transfers to households. To that, the Biden stimulus package (The American Rescue Plan) can be added, amounting to 1.9 trillion US dollars, which corresponds to roughly 9% of the U.S. GDP. The U.S. has also allocated $100 billion to the Endless Frontier Act to promote R&D, innovation and entrepreneurship, targeting 10 areas defined as strategically important. Moreover, a number of European countries have made similar undertakings; France and Germany have earmarked resources for these purposes as well as smaller countries like Finland, Ireland and Norway.

To summarize, there are a vast number of policies on different levels that have been implemented in a more or less ad hoc manner without a consistent strategy or analysis taking into account how these measures interact or whether they complement or counteract each other, i.e., if they generate suboptimal solutions.

## Crisis, stabilization policies, and growth

Crises are recurrent in market-based economic systems and, as noted above, tend to originate in the financial and real estate sectors. Other factors may also generate crises, such as technological paradigm shifts that turn much of the existing know-how obsolete, leading to firm exits, unemployment, and economic hardship. To moderate the fluctuations in economic activity, in particular when the effects are more profound, governments resort to a variety of stabilization policy measures. These have shifted over time, and I will describe the traditionally used measures in the next section and argue why and how they should be complemented with additional instruments.

One particular concern of crises is the possible emergence of long-run hysteresis effects, i.e., persistent negative effects on employment and growth. It may then take considerable time before the pre-crisis growth trajectory and employment participation rates are attained, leading to considerable welfare losses. Furthermore, most crises initiate a restructuring process, opening up new ways of running firms and organizing production, and penetrating markets.^17^ Some industries are threatened, while others will grow and thrive.

In functioning market economies, this will induce a reshuffling of factors of production, and new business opportunities will arise. Such restructuring is likely to be particularly pronounced if paralleled by other profound changes, such as rapid technological development or a globalization process. Hence, economies that can prepare firms and employees for such structural adjustments also stand the best possibilities of reaping the benefits of the ensuing opportunities. Supply-side measures at the micro-level could contribute to facilitating such restructuring, thereby increasing the overall efficiency in moderating cyclical swings, as well as expanding future growth possibilities.

The building blocks of growth are well known: proper institutional conditions regarding the rule of law, well-defined ownership rights, independent and credible courts, the absence of corruption, etc. (Rosenberg & Birdzell, 1986; Acemouglu & Robinson, 2012). Contemporary growth models stress investments in human capital and R&D that are supposed to generate ideas and innovations that then drive, or endogenizes, growth (Aghion & Howitt, 1998; Arrow, 1962; Romer, 1990). More recently, endogenous growth models have been questioned since they neglect the importance of policies that promote the dissemination of new knowledge or new combinations of existing knowledge, to create economic value and societal use.

Two such knowledge diffusing mechanisms that have been highlighted in recent research are entrepreneurship (Acs et al., 2009; Braunerhjelm et al., 2010; Audretsch et al., 2012) and labor mobility (Braunerhjelm et al., 2020; Kaiser et al., 2015). Entrepreneurship, or entrepreneurial human capital, could be seen as a factor of production corresponding to physical or other human capital, which is necessary to unleash business renewal, innovation, and transformation (Audretsch, 2007; Braunerhjelm & Lappi, 2020). Recognizing the role of knowledge diffusion in economic development, which is a considerably more complex policy issue than knowledge investments and encompasses several policy areas (new ventures, taxes, access to capital and competence, competition, etc.), has implications for the design of stabilization policies.

When the economy is exposed to an exogenous shock that affects both demand and supply—as in the case of the current COVID-19 crisis—policy measures to counteract these effects should address both sides. In addition to stimulating demand, policies need to take into account short-run supply-side obstacles that could impede economic recovery while incentivizing activities that could increase an economy’s production potential.^18^ Furthermore, if combined with long-term structural reforms, e.g., with regard to the labor market, infrastructure, housing, and the supply of skills, it is likely to insert confidence within the business sector, which would improve the probability of investments, new ventures, and economic recovery (Stiglitz, 2020). Thus, a partially new and more comprehensive policy design is necessary to cope with crises such as COVID-19.

Conventional stabilization policy tools refer to measures to increase aggregate demand through monetary or fiscal policies (columns 1 and 2, Table 2). The definition of an optimal monetary policy varies from the rather abstract objective of maximizing welfare for a representative consumer given frictions in the economic environment, to the more pragmatic view of minimizing the variance in GDP. Irrespective of definitions, monetary policy has emerged as the primary tool to conduct stabilization policies in recent decades. To achieve this end, central banks have altered the interest rate through open market operations, setting discount rates, quantitative easing, etc., thereby influencing investment and consumption decisions, i.e., demand.^19^ The COVID-19 crisis has also witnessed some unconventional measures, such as the use of helicopter money (direct cash transfers). In addition, monetary policies are responsible for access to liquidity and financial market stabilization. Since the monetary policy has been caught in a liquidity trap situation (zero or close to zero interest rates) for some time, it can be expected to take a more accommodating role, primarily focusing on the latter two tasks.

**Table 2:** An extended framework for stabilization policies

Macro-level Demand-side policies		Micro-level Supply-side policies	
Monetary Conventional 1	Fiscal Conventional 2	Fiscal Unconventional 3	Structural Unconventional 4
Interest rates (open market operations, discount rates, etc.)	Infrastructure projects (roads, digitization, etc.)	Taxes firm level (temporary increased deduction possibilities for investments and VC, temporary abolishment and deferrals, carry back/carry forward, etc.)	Efficiency evaluation of support measures
Provide liquidity	Housing	Furlough wages, subsidies, knowledge upgrading	Support to R&D, innovation activities, new firms
Financial market stability	Tax policies	Subsidies related to reduced revenue, lockdowns, etc	Labor market regulations
Unconventional		State-guaranteed loans, “forgiveness loans”	Competition, regulation of markets
Quantitative easing, helicopter money, etc	Temporarily lowered VAT, etc		Bankruptcy regulations, insolvency

Optimal fiscal policies have been defined similarly, i.e., maximizing the welfare of the representative consumer, traditionally implemented through increased governmental expenditures (e.g., investments in infrastructure, housing). Such policies were common from the 1970s to the 1990s. Some of these measures also improve the supply side of the economy, e.g., by facilitating communications and market accessibility. During the last decades, however, fiscal policies to stabilize the economy have been less frequently applied and more focused on budget balance and sovereign debt ratios.

The definition of an optimal aggregate demand policy implicitly assumes that there are no or few imperfections that hamper or impede the functioning of markets and that transmission to the real economy occurs without frictions. This is rarely the case, however, and these disturbances are frequently associated with institutions at the microeconomic level. The policy measures in columns 3 and 4 (Table 2) address potentially present transmission and supply-side restrictions. These can be considered partly short-term and partly long-term structural measures, where the latter can be expected to instill more confidence among investors and business owners. I will go through these in more detail in the following sections.

As is well known, structural change imposes transition costs as the economy adjusts to new conditions, particularly with regard to increased unemployment and firm closure if exposed to changes in demand and stiffer competition.^20^ Better coordination between demand- and supply-side policies may alleviate such social costs that normally accompany a crisis by enhancing an economy’s flexibility.

### The demand side: monetary policies

More active stabilization policies targeting the demand side of the economy originate in the Keynesian paradigm established in the 1930s, advocating fiscal policies to level out fluctuations in demand. Several decades later, this was challenged by the monetarists’ view (Friedman, 1960; Friedman & Schwartz, 1963) and the proponents that followed, claiming that fiscal policies were inefficient with limited or no long-term real effects. The monetarists emphasized expectations, transparency, and long-term, stable institutions regarding the supply of money. It was argued that monetary policies, at least in the short term, would be more efficient in moderating business cycle volatility.The underlying rationale for this allegation was the relationship captured by the Phillips curve, i.e., a trade-off between inflation and unemployment.^21^ If inflation can be raised by expanding the supply of money, real wages fall while employment increases. However, since the relationship between inflation and unemployment has flattened out over time, the impact of traditional monetary policies can be expected to have diminished also in the short run.^22^

The efficiency of monetary policy has been increasingly questioned, particularly in the aftermath of the extensive measures taken during and after the financial market crisis of 2008–2009 (Mishkin, 2011). The monetary policy then entered into previously unchartered territory, introducing dramatically lower interest rates, even below zero, engaging in quantitative easing that inflated central banks’ balance sheets, introducing forward guidance, and, more recently, attempting to manage the yield curve (keeping long-term interest rates at a given low level).

Monetarists expected that growth would pick up swiftly and economies would return to their former trajectories as those new measures were installed in 2008–2009. This, however, did not happen, even though a lax monetary policy is claimed to have helped cushion the effects of the financial crisis (Jannsen et al., 2015). Rather, the long-term and potentially negative effects on income and wealth distribution, risk exposure of financial institutions investing in high-risk but high-yield assets, regulatory concerns, and the future effectiveness of monetary policy have been emphasized and need further examination.

More generally, Bernanke (2020) stresses that once interest rates hit some lower bound, monetary policies lose much of their edge. The focus should then switch to fiscal policy and vice versa if interest rates are high. Woodford (2020) argues that at low-interest rates, monetary policies fail to stimulate the “right” sort of demand and that fiscal policies may be more efficient when shocks are asymmetric across sectors (as in the COVID-19 crisis), while Bigio et al. (2020) advocate lump-sum transfers when interest rates are at a low level. Additionally, Bagaee and Fahri (2020) stress that monetary policies and non-targeted fiscal policies are less efficient because of sectoral constraints. Research by Levin and Sinha (2020) and Eschenbaum (2019) corroborate those assertions.^23^

A common criticism is that interest rates are not fully transmitted to the real economy, i.e., not affecting consumption, investments, or employment. Boivin et al. (2010) claim that the impact started to decrease already in the 1980s, while Coibion et al. (2020) conclude that lower interest rates have gradually made monetary policies less potent. In the present crisis, it became apparent that policies needed to have a more or less immediate effect due to the fast and drastic collapse of both the supply and demand sides of the economy.

Another new ingredient in monetary policies referred to above is the use of helicopter money (Friedman, 1969). It has primarily been used in the USA, both by distributing a sum of money to each household and through generous temporary unemployment compensations.^24^ Simultaneously, the savings rate has increased in several countries. A potentially more effective version of helicopter money has been suggested by the German economist Gesell (Gesell, 1906). Gesell’s basic idea was also to transfer money to individuals, which would be valid for the procurement of certain goods and services. However, Gesell advocated that the available sum should depreciate over time, meaning that the incentive for consumption in the short run would be much stronger. This mechanism deserves to be considered in the case of rapid and comprehensive falls in aggregate demand.

Hence, the extent to which the giant monetary experiment during the last decade has affected the real economy is debated. Rather, there seems to be evidence of more modest effects on the real economy than initially expected and that the effects appeared with a considerable time lag.^25^

### The demand side: fiscal policies

The infirmity of the monetary policies referred to above has prompted a discussion of whether too much trust in stabilizing the economy was given to that particular measure. In addition, during severe crises, Keynesian fiscal measures tend to reappear, even though the scale has depended on how sizeable budget deficits and sovereign debts were paired with institutional commitments to keep these variables within certain limits. A more balanced use of monetary and fiscal policy measures has been advocated, where the most eager proponents argue that there are no problems with monetizing government debts to finance fiscal expansion.^26^

Fiscal policies have been used quite modestly during crises in the last couple of decades, particularly within the EU, whereas the USA has engaged more actively in implementing fiscal policies. The reasons can be attributed to the conditions outlined in the Maastricht agreement. However, during the present crisis, all former restrictions have been abandoned and fiscal policies have been unprecedentedly extensive in most countries. Globally, fiscal policy measures during 2020 amount to approximately 12% of global GDP, generating an expected global budget deficit of approximately 10–13% and debt levels exceeding 100% (approximately 73% in 2019) of global GDP (IMF, 2020a). This does not preclude the OECD (2020c) from recommending an even more active role for fiscal policies.

Joseph Stiglitz is one of the most eager proponents of fiscal policies to handle unanticipated shocks and structural unemployment, which he argues should also be adopted to combat the COVID-19 crisis (Stiglitz, 2020). Additionally, Blanchard and Summers (2020) believe it is time to rethink the way stabilization policies are used when interest rates are low. They favor increased budget deficits and debt levels at the current low-interest rates, claiming that this will not have any long-term real effects and will be offset by higher growth rates (Eichenbaum, 2019). A similar line of reasoning is pursued by Bianchi et al. (2020), who propose setting up an “emergency” budget combined with a prolonged period of inflation below the target to remedy the COVID-19 crisis.

Traditionally, fiscal policies have involved large infrastructural projects, such as transport and housing, intending to increase employment and positively affect aggregate demand. Due to considerable time lags, these projects have often become pro-cyclical, implying that once these often complex projects have been initiated, the economy has already entered an upward stage of the business cycle (Bianchi et al., 2020). Moreover, Ricardian equivalence and consumers’ perception of a permanent income over their life cycle suggested that the expected effects of fiscal policies were considered limited (Lagerwall, 2019).^27^

Proponents of fiscal policies assert that a multiplier effect of 2.5–3, i.e., each euro or dollar spent could increase economic activity by another three (IMF, 2020), can be expected for adequately designed policies. There is also an increasing group of scholars recommending fiscal policies to target long-term structural problems simultaneously as short-term incentives are introduced to increase consumption and investment to accelerate growth (IMF, 2017; Borio & Hofmann, 2017; Yülek et al., 2020).

### The supply side: microeconomic measures

The supply side of the economy refers to start-ups, growing incumbents, investments, supply of labor, access to capital, and competence together with technological progress and innovation. Entrepreneurs, bringing forward new products and services and identifying new business models, markets and inputs, are of particular interest.^28^ They are critically important for creative destruction processes that, in turn, are imperative for the renewal and transformation of an economy that follows a crisis.^29^ These processes can be supported through several policy instruments, although not primarily with traditional stabilization policy tools. Neither negative interest rates nor infrastructure projects create societal value per se; the opportunities generated through these measures have to be converted into economic activities by individuals and firms, which requires a dynamic business sector. Hence, supply-side measures can be expected to complement and reinforce traditional stabilization policies.

According to real business cycle theories, supply shocks could stem from technological change, hikes in prices of raw material (e.g., oil), natural disasters, or pandemics (Kydland & Prescott, 1982). Implementing traditional monetary and fiscal policies to increase aggregate demand will, then, not be efficient, and the supply side should be targeted directly to overcome constraints.

Stiglitz (2020) emphasizes the importance of supply-side measures when a crisis generates sectoral unemployment of capital and labor (as in the present crisis). When sectors are affected differently, the Keynesian multipliers may be partially muted, and traditional policies to stimulate demand may be less effective. Instead, more discretionary policies are claimed to be more efficient, e.g., subsidizing unemployed individuals and providing access to finance for firms or sectors (Guerrieri et al., 2020). Still, these remedies do not deviate dramatically from traditional fiscal demand measures.^30^

More generally, supply-side policies should aim at providing support and incentive structures that encourage individuals and companies to start firms, experiment, grow, and invest in new ideas. When a crisis strikes, particularly when it is beyond what firms or individuals can influence or anticipate, threatening the survival of a large number of firms or even industries, different types of discretionary micro-level policy interventions targeting different areas may be warranted. The appropriate measure depends on the depth and extent of a crisis and how symmetric the effects are. In cases where a crisis is reinforced by policy decisions that hinder or basically ban certain types of commercial activities, it could be argued that policymakers have a strict responsibility to bridge such economic disturbances. Examples in the current crisis would be firms in the traveling, tourism, events, and entertainment industries.

During the COVID-19 crisis, rather unorthodox microeconomic policy measures directed toward the supply side of the economy were introduced. Tax and payroll fee deferrals have been common, even tax exemptions for certain periods and industries. Additionally, different kinds of direct financial support to secure firms’ survival have been used, as well as state-guaranteed loans and subsidized interest rates. These policy experiments constitute a testbed of different interventions from which important lessons can be drawn.

## Realigning macroeconomic and microeconomic measures

The importance of coordinating traditional monetary and fiscal policy measures to stabilize the economy has long been underscored in the literature.Woodford and Xie (2020) find that the efficiency of fiscal policies is contingent upon an accommodating monetary regime. Otherwise, fiscal policies may trigger hikes in interest rates and vice versa, leading to suboptimal stabilization policies according to Bianchi and Melosi (2017).^31^ They furthermore conclude that it is necessary to consider both long-term and short-term policy targets in a crisis situation, e.g., with regard to inflation levels or debt levels. Benmelech and Tzur-llan (2020) note that the possibility of combining monetary and fiscal policies is not feasible for all countries but depends on sovereign debt and credit rankings, i.e., there is a limit to the extent that fiscal policies can be implemented. In particular, poorer countries with a sizeable debt burden may be stripped of the opportunity to use more efficient fiscal policies and have to resort to monetary policies.

Even though there seems to be a consensus that a concerted strategy of stabilization policies to stimulate the demand side of the economy is needed, a more systematic inclusion of the supply side has not been considered to the same extent. In particular, the issue of how to integrate microeconomic supply measures to stabilize an economy is basically neglected in the policy discussion. Giving the supply side of the economy a more prominent role in stabilization policies requires the presence of an entrepreneurial business sector, i.e., economic agents—individuals, start-ups, and incumbents—that will react and convert policies into economic activity.

Moreover, as suggested in the knowledge spillover theory of entrepreneurship, different types of firms can be expected to contribute differently to economic dynamism (Acs et al., 2009; Braunerhjelm et al., 2010). Additionally, in the present context, I stress the importance of addressing both new and young firms as well as older incumbents. The former are often more risk-prone and experimental; they tend to drive innovation and competition, but they are also more vulnerable and exit prone. Incumbents are better at scaling up operations, making gradual improvements and innovations and reaching out internationally (Baumol, 2002; Christensen, 1997). By including supply-side measures at the micro-level, firms’ enhanced possibility to survive, innovate, and scale-up also improves the scope for recovery. Such supply measures may also create demand, for instance, by innovative firms providing cheaper goods and services in an economic downturn.^32^

Several of the proposed measures have been implemented previously, whereas others are new. To affect the business cycle, interventions should have a temporal, short-term perspective. However, their efficiency depends on an appropriate long-term institutional framework to promote market dynamics. Hence, I will consider both short-term interventions and necessary long-term reforms where the overarching objectives of policy should aim at increasing industrial dynamism and to provide possibilities for firms and industries to strengthen their long-run competitiveness.

Before delving into the suggested micro-level policies, the importance of having well-defined objectives with policy interventions and measurable criteria, followed by stringent evaluations of whether the stated objectives have been attained, should be stressed. There may be strong theoretical arguments to undertake different policy measures, yet the empirical underpinnings are presently scarce or nonexistent.^33^ Therefore, well-designed efficiency evaluations of interventions are crucial to learn and adapt policies and to allocate scarce funds to those measures that prove to be most effective.^34^ Inefficient support schemes and subsidies should be phased out.

Four micro-level policy areas (knowledge upgrading, taxes, financing, and the labor market) are argued to be particularly important vehicles to combat crises and moderate swings in the business cycle but also to strengthen the growth potential and build resilience against future crises. Finally, I will discuss the importance of institutions that generate competitive markets and proportionate insolvency regulations that does not stigmatize failures, both of which are long-term necessary conditions for adaptive and dynamic markets.

### Knowledge upgrading: part of support schemes

During the financial market crisis of 2008–2009, the German system of Kurzarbeit—implying that the government stood for approximately 60% of the wage costs for employees whose working hours were reduced—was viewed as a successful tool to retain competence within a firm and keep unemployment at low levels (IMF, 2020b). Other countries, e.g., Finland and Sweden, introduced differentiated versions of Kurzarbeit systems. Training possibilities were not compulsory, albeit sometimes they were offered, and firms were encouraged to engage in knowledge-upgrading activities. During the current crisis, there seem to be more initiatives and support to engage employees in educational activities, such as programming and digitization (Eurofond, 2020).

Engaging employees in knowledge-upgrading schemes, while they are subsidized by governmental funding, appears to be a logical extension of the Kurzarbeit system. More precisely, access to support schemes should be conditioned on managers and employees agreeing to engage in knowledge-enhancing activities. This would facilitate a quick rebound when the market turns, putting less strain on structural adjustments and strengthen countries’ comparative advantages in knowledge-intensive production. Moreover, assuming that knowledge and its diffusion are the critical ingredients, this would expand the potential for future economic growth.

The ambition should be to improve knowledge in areas where gaps are likely to be greatest; digitization, innovation, sustainable production systems, and resilient internationalization are obvious candidates.^35^ Hence, this could be a way to close the digital gap claimed to exist between primarily SMEs and larger firms (OECD, 2020d). The productivity potential of acquiring knowledge related to artificial intelligence, the internet of things, automated production processes, etc., is huge, particularly for SMEs.

Most countries have specific authorities that administer policies directed toward the business sector. Their responsibilities could be extended to include training activities, e.g., by providing links to online educational programs carried out by digitized education companies, universities and other higher education institutions, or other firms. These services could be procured by the governmental authorities or by firms directly through a voucher system.

It seems reasonable that governments take the major brunt of the costs associated with knowledge upgrading due to externalities likely to benefit society as such. Nevertheless, firms and employees should also bear part of the costs to incentivize their choices and participation.

### Tax: corporate automatic stabilizers and incentives

Taxes are perhaps the most potent microeconomic policy instrument to which politicians have access. However, taxes are often reduced to a tool for redistribution. The level and structure of taxes govern the behavior of individuals and companies; education, investment, sustainability, risk-taking, and the supply of labor are all affected by the tax system. Also, social norms and the legitimacy of the tax system are anchored in its design and level.

Taxes should be designed such that more “corporate automatic stabilizers” are at work that would complement automatic stabilizers related to social insurance systems and unemployment compensations. Presently, the state claims a slice of profits made by firms through corporate taxes. In a more symmetric structure, the state would also participate in the sharing of losses, which would strengthen the financial position and reduce liquidity constraints when firms are exposed to severe economic shocks. Such systems are in place primarily in Anglo-Saxon countries, where they can be extended during crises (as was the case in the U.S. 2008–2009). A conceivable structure would allow current losses to be offset against profits during, for instance, a 3- to 5-year period (accumulated or as an average), i.e., a carry-back system. Dramatic downturns in a firm’s market would then be cushioned by an automatic refund from the state.

Similarly, postponing taxes by allowing a share of profit to be reserved for future use would also help in times of crisis-induced liquidity constraints. Such reservation possibilities are in place in most developed economies, but their efficiency depends on the number of years such reserves can be retained and the allowed amount.

Targeted and temporary tax incentives could also be implemented to smooth out swings in investment activity. Investments are expected to fall between 15 and 25% in 2020 within the EU (European Commission, 2020), further magnified by an additional expected 40% reduction in foreign direct investment according to Unctad (2020). Decreased investments risk further accentuating the lackluster productivity performance witnessed among OECD countries during the last decade (OECD, 2019). To offset a decline in investments, more generous depreciation possibilities could be allowed for a limited amount of time, e.g., 24 months.^36^ Similar measures could be undertaken to stimulate innovation, i.e., temporary and more generous possibilities to deduct R&D expenses from revenue, taxes, or payroll fees. As mentioned above, a number of countries have introduced temporary reliefs on payroll costs of employees combined with furlough wages, which facilitates keeping the supply side of the economy intact.

The above measures target incumbents. To safeguard and promote entrepreneurial endeavors, policies could encourage early-stage investments through a time-limited “superinvestor deduction” possibility directed toward business angels and other early-stage investors. As shown by Howell et al. (2020) in a study on crises in the USA since the 1970s, early-stage investors tend to withdraw to later stages with negative effects on innovation quality. In the somewhat longer run, such incentive measures must be combined with less restrictive policies on the possibilities to issue employee stock options (present restrictions are associated with sectors, full-time requirements, salary thresholds, etc.). Scaling up businesses and attracting adequate competencies to new and young firms require a competitive stock option facility.

### Financing: targeting the right recipients

In several countries, the increase in lending to business over the last decade has, to a large extent, not reached young and small firms. Instead, the overwhelming part has gone to large firms and real estate companies. This is linked to the international regulatory framework (Basel IV) of financial markets, with specific rules for risk assessments and collateral requirements.^37^

To reduce financial constraints during the COVID-19 crisis, a large number of countries have introduced state guarantees on bank loans, primarily directed to SMEs. In the USA, the PPP schedule referred to above seems to be an appropriate measure, considering the harsh conditions on loans given to SMEs (Chodorow-Reich et al., 2020; Hubbard & Strain, 2020). They include so-called “forgiveness loans,” implying that loans were written off for firms that maintained their labor force, which seems to have worked reasonably well (OECD, 2020b). In comparison, state-guaranteed loans provided through commercial banks that undertake an ordinary credit assessment process seem to have fared worse. Together with the guarantee frequently being subject to an administration fee, a large share of SMEs are effectively excluded from this facility.^38^

To efficiently provide funding during severe downturns in the economy, state-guaranteed loans are motivated, but these must be appropriately designed to reach those in need. First, guarantees should be maximized in accordance with financial regulations (e.g., currently 90% within the EU over a credit period of 6 years), while admission fees should either be abolished or differentiated depending on the size of the firm. If the bank guarantee is set at a too low level, there might be incentives for banks to claim companies in bankruptcy when the time for the termination of the guaranteed loan approaches. After the guarantee period has elapsed, the possibility of converting such loans to long-term credit facilities with a maturing time of 15–20 years, but at market rates, should be considered.

In addition, the supply of venture capital seems to have diminished during the crisis (Gompers et al., 2020), which has led some countries to introduce public venture capital funds. This could complement the tax incentives discussed in Sect. 4.2 and be organized such that state-backed funds syndicate with private investors.^39^

### Labor market: mobility and skills

Furlough schemes and other subsidies to retain employees have the advantage of firms being able to pick up production on a short notice once the economy turns upward. If those firms instead had closed down, it would take considerable time (if ever) to replace them with new firms. Nevertheless, if furlough schemes become long term, they may have an adverse effect on an economy’s ability to adjust and restructure. The introduction of new business models, new firms, and innovations may be stalled, and the appearance of so-called zombie firms could increase, tying up financial and real resources. Thus, there is a trade-off between direct and indirect costs related to furloughs and the negative effects of firms permanently closing down.

Several OECD studies have identified labor market regulations as being one of the most resource consuming and costly regulatory areas (OECD, 2020e). In addition, there are other negative supply-side effects related to labor market regulations. A previously overlooked but powerful mechanism for increased innovation appears to be flexible labor markets characterized by high mobility (Braunerhjelm et al., 2020; Kaiser et al., 2015). The positive association between innovation and labor market flexibility is attributed to a better matching of competencies and broadened knowledge networks and is also shown to positively influence intrapreneurship in SMEs (Braunerhjelm et al., 2018).^40^ Improving mobility and matching should be considered a long-run objective, complementing short-run initiatives to enable firms to retain their employees.

### Long-term key policies: competition and insolvency

Two interlinked features of dynamic markets are the level of competition and well-balanced insolvency roles such that business failures do not stigmatize owners and entrepreneurs. Both policies are important long-run conditions for effective stabilization policies targeting the supply side of the economy.

There is an obvious risk that massive interventions to provide support at the micro-level may end up in new regulations and support structures with negative effects for competition. In addition, the last few decades have witnessed the emergence of digitized production technologies and new business models, a trend that has been reinforced during the present crisis. A conspicuous phenomenon is the emergence of platform companies, often global, which have had considerable positive consumer effects in the short run, while the long-run welfare effects are uncertain and potentially negative (Cremér et al., 2019).

The advantage of first-mover advantages, network externalities, low marginal costs, and access to large amounts of data implies that there is only room for a few dominant players in the respective industry where they are present. The increase in market concentration is particularly accentuated in the USA and embraces most industries (Phillipon, 2019), but other countries have also experienced the same trend. Likewise, a decline in new ventures has been observed in several countries, particularly in the USA (Naude, 2019), adding to the worries of languishing economic dynamism.

The current framework on competition is based on how the abuse of market power, mergers of firms (given certain turnover thresholds), and different types of collusions (prices in particular) may harm consumers by raising prices. In the platform economy, it may be considerably more intricate to verify that harm has been inflicted upon consumers, especially in the short run, since services are often free. The skepticism related to platforms concerns their long-term impact on competition, innovation, and entry. The potentially negative effects have been increasingly emphasized and have sparked efforts to adjust institutions to incorporate the specific characteristics of platform firms and their potential future impact on industrial dynamics in both the EU and the USA.^41^ Additionally, individual countries need to modify the national legal framework institutions to avoid stifling entry, innovation, and consumer welfare.

Another cornerstone of dynamic and adaptive markets refers to insolvency institutions. During a crisis, firms will experience insolvency problems, particularly when the demand and supply sides are affected simultaneously. A number of countries have taken measures during the crisis to avoid bankruptcies on a large scale, including temporary moratoriums. At the same time, the crisis involves a reshuffling of factors of production from low-productivity to high-productivity firms. This is an essential part of the creative destruction process.

As has been shown by Eberhart et al. (2017), the quality of insolvency rules—bankruptcy and reconstruction—also affects the quality of start-ups, as well as the attitude toward risk. Entrepreneurs and incumbents who find themselves in insolvency problems must be given a reasonable possibility of reconstructing or, if bankruptcy is unavoidable, to not be burdened for life. Properly designed insolvency regulations thus have the potential to cushion the effects of a crisis and contribute to an efficient reallocation of resources.

The proportions of firms going bankrupt and those entering a reconstruction process depend on the regulatory design. If the bankruptcy alternative means lower risks in terms of future debts and tax liabilities for owners, management teams, and board members, even though firms may be in temporary financial difficulties, it may lead to an “excessive” number of exits of firms. As argued by Panizza (2020), a regulatory framework implying that bankruptcy is preferred over other alternatives exacerbates the risk of widespread unemployment and that financial markets become affected.

## Conclusion

Based on a literature review of stabilization policies during primarily the two latest crises—the Great Recession of 2008–2009 and the ongoing COVID-19 that started in 2020—and the plethora of policy measures implemented during 2020, I have argued that the traditional toolbox used to stabilize fluctuations in an economic activity needs to be supplemented. The reasons can be summarized as follows.

First, monetary policy has lost much of its clout due to already exceptionally low-interest rates, even though the important tasks of securing financial market stability and access to liquidity remain. Simultaneously, acceptance of fiscal policies has increased, triggered by low-interest rates. However, the level of interest rates may change quite quickly in the wake of the massive fiscal stimulus packages to be undertaken in particularly the USA and to some extent within the EU. This may constrain countries that are already struggling with high levels of debt and sizeable budget deficits.

Second, the supply side of the economy has basically long been neglected in stabilization policies. The standard measures of large-scale infrastructure investments and similar projects were previously undertaken quite frequently and are presently facing renewed interest focusing on environmental projects, infrastructure maintenance, etc. However, the aim should be to increase demand while simultaneously taking into account supply-side factors, combined with a coherent realignment of policies at the micro- and macro-levels.

Third, even though some of the policies undertaken during the present crisis have been used previously, there are a host of unconventional measures that are more or less experimentally implemented with uncertain effects. Subsidies directed toward firms and employees have flourished. A guiding principle should be that access to subsidies is conditioned on firms’ and employees’ participation in knowledge-upgrading activities. Overall, the testbed of policy measures needs to be rigorously evaluated to filter out the most efficient tools to ameliorate the capacity to handle future crises.

Hence, the main conclusion is that an extended stabilization policy framework should incorporate policy measures that target the supply side of the economy. Temporary policies that aim at smoothing out cyclical effects at the firm level, including investments in real and human capital, access to finance, etc., should be combined with long-term reforms to instill confidence among economic agents regarding future market opportunities. Such measures would serve the dual purpose of strengthening the growth potential over time (augmenting knowledge) and lowering the social costs that appear as economies enter a period of structural adjustment, e.g., higher unemployment (training of employees, facilitating the entry of firms, etc.). This would enhance the business sector’s future competitiveness and strengthen the knowledge base of an economy.

A further effect may be to partially endogenize an economy’s ability to stabilize economic activity as it is exposed to shocks, i.e., make it more resilient. That is however an issue that would require more space than presently available and is a topic for future research.

## Footnotes

1 This article partly builds on my work for the Swedish Re-Start Commission, organized by the Stockholm Chamber of Commerce (Braunerhjelm 2020).

2 The rate of vaccination has increased in early 2021 which should insert confidence in the business sector, facilitate mobility, and enable employees to get back to work, thereby also accelerating recovery. However, how efficient the vaccine is against new mutated versions is still unknown.

3 Global value-added chains slowed down somewhat after the 2008–2009 crisis but then started to grow again, albeit at a slower rate. After 2017, growth seems to have picked up until the 2020 crisis (OECD 2020a). Regarding migration, data is lacking for the most recent years, but flows have been increasing for some time (see Migration Data Portal, 2020). The projection of the global population increase varies, but there is no doubt that it will grow (World Population Stats, 2021).

4 These interdependencies between the demand and supply side from a stabilization policy point of view have long been noted. Guerrieri et al. (2020) looks at the transmission from the supply side to the demand side in the present crisis, see also Fornaro & Wolf (2020).

5 The requirements that budget deficits should not exceed 3% of GDP and governmental debts at 60% of GDP have been abolished until 2023. Presently, there are discussions about when exactly these will be re-imposed and the extent to which they will be modified.

6 The subsequent sections also entail a discussion of the efficiency of traditional monetary and fiscal policies, improved coordination of monetary and fiscal policies and the possibility of using other instruments to stimulate aggregate demand.

7 See Baldwin and Weder di Mauro (2020) and Bénassy-Quéré and Weder di Mauro (2020) for an account of policies comprising a large number of areas.

8 In a recent discussion regarding the EU’s recovery fund (750 billion EUR) with representatives from the Commission, it became clear that growth aspects were absent from the prerequisites to obtain means from the fund.

9 Indebted businesses, being dependent on creditors or subsidies for their continued existence, are effectively put on never-ending life support while tying up resources such as labor and capital.

10 According to Hepburn et al. (2020), climate investments are associated with particularly large fiscal policy multipliers.

11 Based on a large number of interviews with business representatives in Sweden, and initiatives such as the Science Based Target initiative (comprising 150 large global companies), there appears to be a strong commitment from the business community to undertake environmental measures in combatting the crisis. Only 4% of the EU’s various action proposals are estimated to promote climate improvement (Hepburn et al., 2020).

12 Note that the crisis is symmetrical insofar that it cannot be linked to a particular industry (as in the 2008–2009 financial crisis) or an individual country. However, its effects are asymmetrical. In other words, it strikes differently across countries and industries depending on labor intensity, supplier structures and specialization.

13 The Economist (2020).

14 See, e.g., Eurostat (2020b).

15 Some main features are to allow direct capital injections to companies (up to 800,000 euros per company), loans with subsidized interest rates (6 years), government-guaranteed loans (90%), deferral of taxes and payroll fees, and wage subsidies (redundancy/furlough wages). The conditions are that companies receiving support have had a financially sound position in 2019 that loans and guarantees do not exceed twice the wage cost or 25% of turnover and that liquidity strengthening measures apply for a maximum of 18 months for SMEs and 12 months for larger business.

16 In some cases, they seem so generous that they are likely to violate the revised state aid rules that now apply (OECD, 2020b).

17 This is quite obvious from previous crises such as the oil price shocks in the 1970s, the financial market deregulation in the 1980s, the real estate crisis in the 1990s, and the dotcom and the financial market crises in the 2000s.

18 There is a huge literature on stabilization policies, see, e.g., Mishkin (2006) and Woodford (2010) for standard presentations of monetary strategies and optimal policies. See also Lagerwall (2019) for a survey of the reassessment of macroeconomic stabilization policies and VoxEU & CEPR Coverage of the Covid-19 Global Pandemic (voxeu.org).

19 Given their mandate in terms of the rate of inflation end economic stability the level of interest rates that central banks target is often based on the Taylor rule, i.e., the inflation rate, real GDP, and the output gap.

20 See for instance Cacciatore et al. (2015), who, using a dynamic stochastic general equilibrium model, conclude that deregulating the labor and product markets will generate costs in the short run.

21 The Phillips curve is related to the quantity theory of money which according to monetarists implies that an increase in the supply of money will increase prices (for a given volume of transactions) since the turnover (velocity) of money is assumed constant. Keynesians criticize the assumption of constant velocity and price flexibility, claiming that an expansion of the money supply would instead affect interest rates and investments.

22 Moreover, if rational expectations prevail (Lucas 1972; 1976), increasing the supply of money would have no real effects even in the short run, except for increasing inflation. Barro (1976) pursued a similar fiscal policy argument, i.e., it would be inefficient due to Ricardian equivalence. The neo-Keynesian approach, incorporating much of the monetarist view, however, claimed that monetary policy would have effects due to bounded rationality, inflexible prices, and wages (Bradford de Long, 2000). Hence, monetary policies would have effects at least in the short run.

23 Taylor (2017a), on the other hand, strongly advocates a systematic rule-based approach compared to more discretionary policies.

24 Unemployment compensation exists in all EU countries but the level varies. The USA generally has lower levels and coverage of unemployment insurance but has introduced financial compensations that exceed the minimum wages during the first stage of the crisis.

25 This has led to a discussion whether the current targets of monetary policies, primarily the inflation level in the short to medium term, is appropriate or if alternative targets such as the range within inflation should be allowed to fluctuate, nominal targets, average inflation in the longer run, etc., should be considered. The USA has recently altered its inflation target which is now defined as 2% on average over a longer time period.

26 This adheres to the so-called modern monetary theory, claiming that there is basically no restrictions for expansive fiscal policies to be financed by printing money. This is a highly discussed and questioned theory that will not be considered henceforth, see also Borio and Hofmann (2017).

27 Another line of criticism claims that fiscal policies, for instance by introducing new welfare schemes or subsidies, correlate better with elections than the business cycle (Blais & Nadeau, 1992).

28 For example, AliBaba, Amazon, Über, AirBnB, and Spotify all started during crises. There is no clear indication of whether innovative firms are more or less resilient to crises (Williams et al., 2017).

29 Studies examining how start-ups are affected by the business cycle are ambiguous. Klapper and Love (2011) find a pro-cyclical effect whereas Paulson and Townsend (2005) report a countercyclical effect. Bradley et al. (2011) conclude that young and small firms are less negatively affected by crises. Others find a decline in start-up activity, at least in the beginning of a downturn, but also that start-ups precede a more general upswing in economic activity (Koellinger & Thurik, 2012; Parker, 2012). See also Congregado et al. (2011) and Gonzaléz-Pernia et al. (2018).

30 Bordo and Duca (2020) show that the unconventional initiative by central banks to purchase corporate bonds has contributed to lower interest rates and led to a less exposed financial situation for firms, thereby improving supply-side conditions and dampening the negative macroeconomic effects.

31 The mechanism of uncoordinated policies implies that an expansionary fiscal policy that may generate higher inflation induces monetary policies that try to stifle higher price levels by raising interest rates which may in turn hamper growth, etc., see Stupak (2019) and IMF (2017) for a brief review of these mechanisms.

32 That is basically the idea behind AirBnB and Über; both started during the financial crisis of 2008–2009. Hence, even if Say’s law (Say, 1803) does not pertain at the aggregate level or to the majority of sectors within an economy, it might be valid for some industries.

33 This is comparable to the situation before quantitative easing was introduced.

34 Efficiency evaluations can be defined as calculating the social and private welfare effects of policy interventions. Infrastructure projects are often subject to careful cost–benefit analysis from a societal welfare perspective. Different competing infrastructure projects are then ranked according to their expected benefits, which also govern the investment decisions. A similar procedure is, however, also possible to evaluate other policy projects. Process evaluation, focusing on the administration of the support for the provider and recipient, is much more common. For a survey of the challenges related to policy evaluations, see Bovens et al. (2009).

35 Even though globalization can be expected to slow down in the short run, differences in production and transportation costs are likely to determine the long-run production structures.

36 A “superdeduction” possibility has recently been introduced in the UK, amounting to 135% of the investment costs (https://www.gov.uk/guidance/super-deduction).

37 See for instance McKinsey & Company (2020), claiming that most bank risks are related to SME loans.

38 It varies across countries, but several report low usage of guaranteed loans.

39 Andersson et al. (2020) suggest a large European equity fund in order to avoid distortions across EU countries due to different financial strengths. The fund is proposed to operate under the control of EIB.

40 Digitization has recently challenged existing labor market regulations through the emergence of the gig and sharing economy. This has led to a legal discussion whether gig workers should be defined as employees, self-employed, or perhaps as a third category (“dependent workers”), see Taylor (2017b). A completely new category in the labor market—the “self-hired”—has also emerged, which is a kind of hybrid between self-employment and being employed. Recent court decisions in the UK and the USA suggest that gig workers’ rights have become more similar to those of regular employees.

41 See Crémer et al. (2019) and Scott Morton et al. (2019).
